# Supplementary material for: A single spin in hexagonal boron nitride for vectorial quantum magnetometry
Source: Nat Commun. 2025 May 28;16:4947. doi: 10.1038/s41467-025-59642-0 (PMC12119844; doi:10.1038/s41467-025-59642-0)
Supplement: Supplementary file 1 — Supplementary Information [file 41467_2025_59642_MOESM1_ESM.pdf]

Supplementary Information for: A single spin in hexagonal boron nitride for vectorial quantum magnetometry

Carmem M. Gilardoni,[1,2,*,†] Simone Eizagirre Barker,[1,*] Catherine L. Curtin,[1] Stephanie A. Fraser,[1] Oliver. F.J. Powell,[1,3] Dillon K. Lewis,[1] Xiaoxi Deng,[1] Andrew J. Ramsay,[3] Sonachand Adhikari,[4] Chi Li,[5,6] Igor Aharonovich,[5,6] Hark Hoe Tan,[4] Mete Atatüre,[1] Hannah L. Stern[7,†]

[1] *Cavendish Laboratory, JJ Thomson Avenue, University of Cambridge, Cambridge CB3 0HE, UK*

[2] *Centro Brasileiro de Pesquisas Físicas, Rua Dr. Xavier Sigaud 150, Rio de Janeiro 22290-180, Brazil*

[3] *Hitachi Cambridge Laboratory, Hitachi Europe Ltd., JJ Thomson Avenue, Cambridge CB3 0HE, UK*

[4] *ARC Centre of Excellence for Transformative Meta-Optical Systems, Department of Electronic Materials Engineering, Research School of Physics, The Australian National University, Canberra, ACT 2600, Australia*

[5] *ARC Centre of Excellence for Transformative Meta-Optical Systems, Faculty of Science, University of Technology Sydney, Ultimo, New South Wales, Australia*

[6] *School of Mathematical and Physical Sciences, Faculty of Science, University of Technology Sydney, Ultimo, New South Wales, Australia*

[7] *Department of Materials, University of Oxford, Parks Road, Oxford OX1 3PH, UK*

Version of April 15, 2025

* CMG and SEB contributed equally to this work

† Correspondence to CMG at gilardonicm@cbpf.br or HLS at hannah.stern@materials.ox.ac.uk.

CONTENTS

Supplementary Note 1. Variations in cwODMR contrast between defects 3

Supplementary Note 2. PL saturation and comparison with the NV centre 5

Supplementary Note 3. Rabi measurement 6

Supplementary Note 4. Background correction of intensity autocorrelation measurements 7

Supplementary Note 5. Kinetic model of the system 8

A. $g^{(2)}(t)$ experiments 10

B. Spin-dependent initialisation experiment 11

C. Modified spin-relaxation experiment 12

D. cwODMR contrast 13

E. Fitting Procedure 14

F. Uncertainties in Estimated Parameters 15

Supplementary Note 6. Comparison between models with *S=1* in ground state or metastable state 18

Supplementary Note 7. pODMR and $g^2(t)$ data with associated global fits for additional defects 21

Supplementary Note 8. cwODMR spectra 24

A. cwODMR spectra associated with Figure 2e 24

B. cwODMR spectra associated with Figure 3a 24

C. cwODMR spectra associated with Figure 3c 28

Supplementary Note 9. Excited state zero-field splitting parameters 30

Supplementary Note 10. Sensitivity Range 32

References 33

Supplementary Note 1. VARIATIONS IN CWODMR CONTRAST BETWEEN DEFECTS

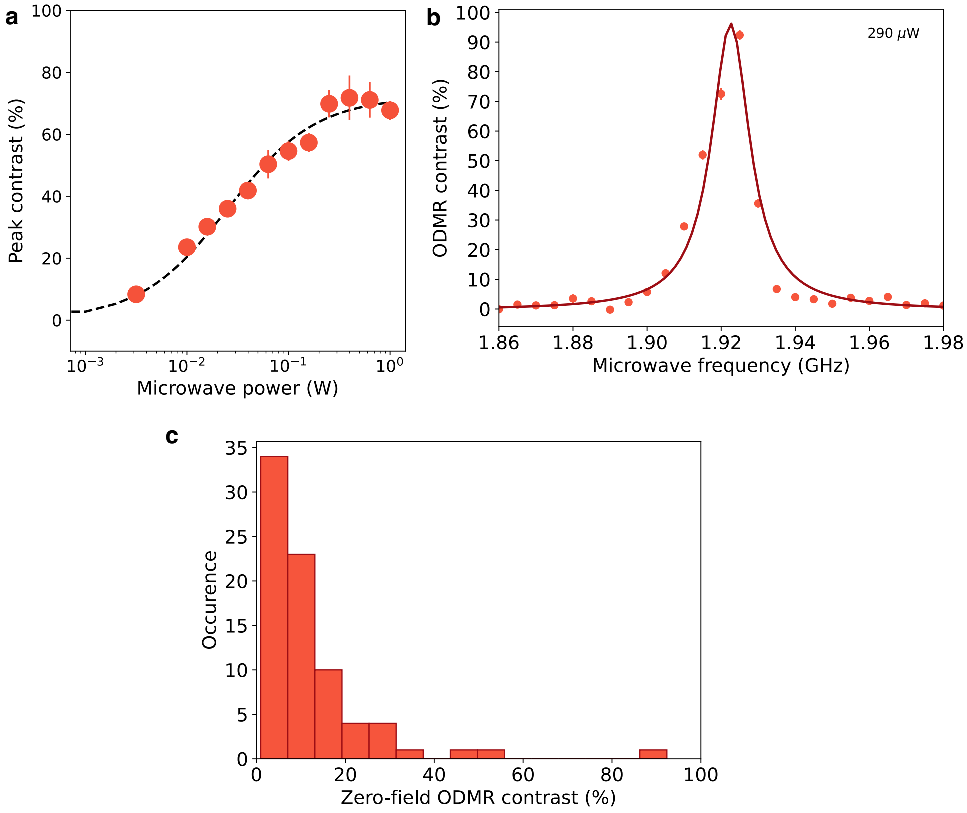

Supplementary Fig. 1. **Variations in zero-field contrast across hBN defects.** (a) cwODMR contrast f_B resonance as a function of microwave power showing clear saturation behaviour, for a defect illuminated with 150 μW optical power. (b) cwODMR spectrum of the f_B resonance same defect, taken highest maximum microwave (1.5 mW) and optical powers accessible (290 μW). The contrast reaches >90%. (c) Histogram of the maximum cwODMR contrast observed for 79 defects in the absence of a magnetic field. These measurements were taken at 1.5 mW microwave power.

The saturated cwODMR contrast varies across defects, ranging from 1-2% to over 90%. In Supplementary Fig. 1a, the peak cwODMR contrast is presented as a function of microwave power, exhibiting clear saturation behaviour. In Supplementary Fig. 1b, the cwODMR contrast of the f_B resonance of this defect reaches >90%. The distribution of saturated contrast across defects is represented by the histogram in Supplementary Fig. 1c. These values correspond to peak cwODMR contrast of the f_B resonance, measured under microwave saturation conditions. However, a full laser power-dependence of the contrast was not conducted for all defects presented, such that the value of contrast quoted here presents a lower bound.

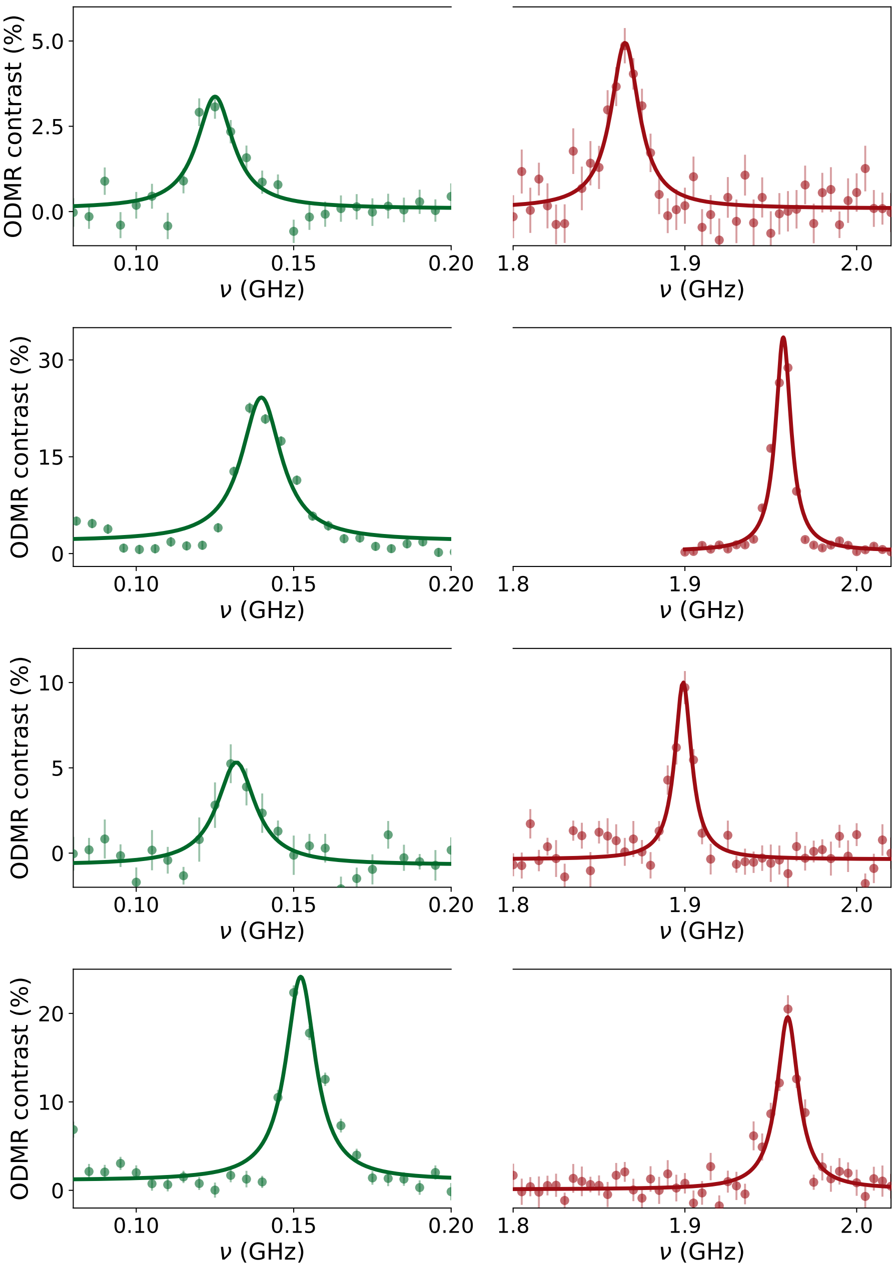

Supplementary Fig. 2. **Saturated cwODMR spectra of four defects at zero magnetic field.** For these defects, the contrast of f_{A} is comparable to that of f_{B}, while the contrast of f_{C} is lower than the signal-to-noise ratio of the measurement.

The relative distribution of contrast between the three possible cwODMR resonances f_{A}, f_{B}, and f_{C} is dependent on the defect. For all defects, we observe the highest magnitude of contrast into the f_{B} resonance. For many, we see comparable magnitude between f_{A} and f_{B} (as shown in Supplementary Fig. 2), while for others contrast is more evenly distributed between f_{A} and f_{C}.

Supplementary Note 2. PL SATURATION AND COMPARISON WITH THE NV CENTRE

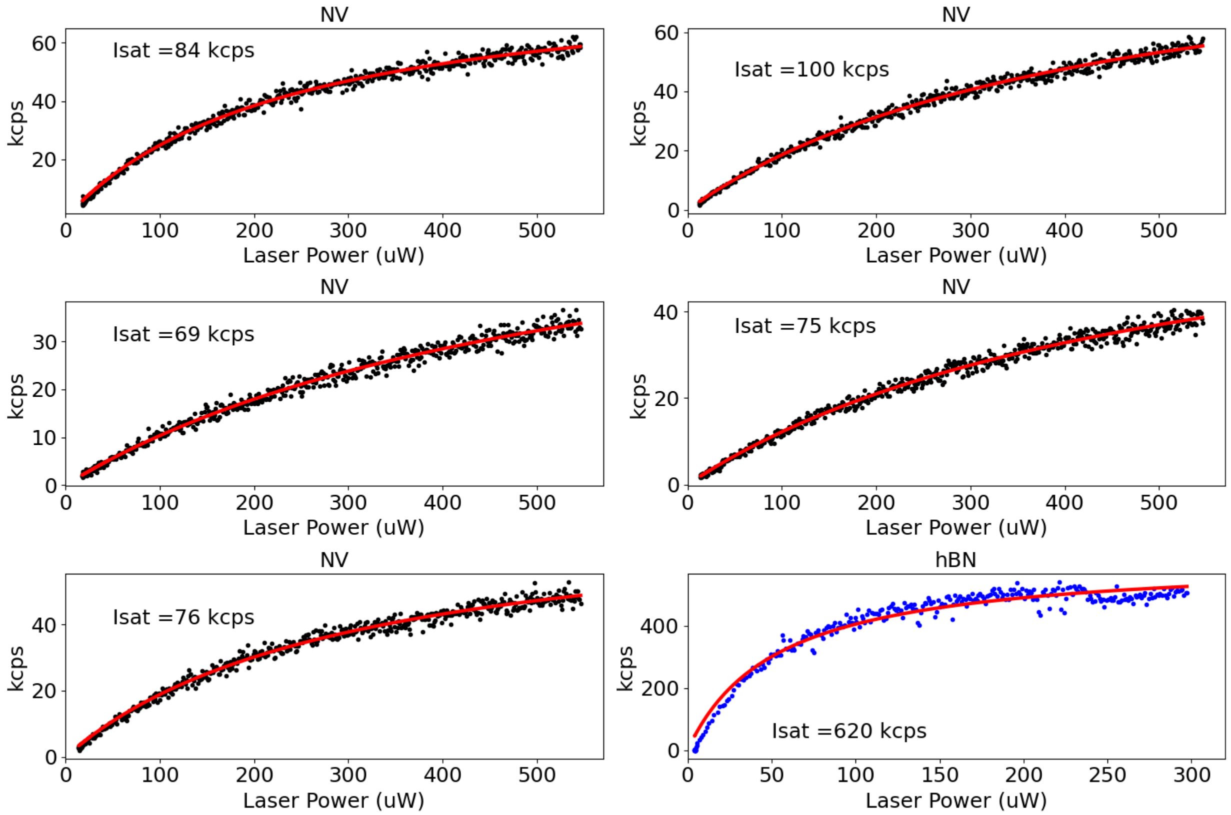

Supplementary Fig. 3. **Comparison of PL at saturation between NV centres and hBN defects.** Example saturation curves measured for single NVs embedded in nanodiamonds and on a single hBN defect, in the same experimental setup.

Supplementary Note 3. RABI MEASUREMENT

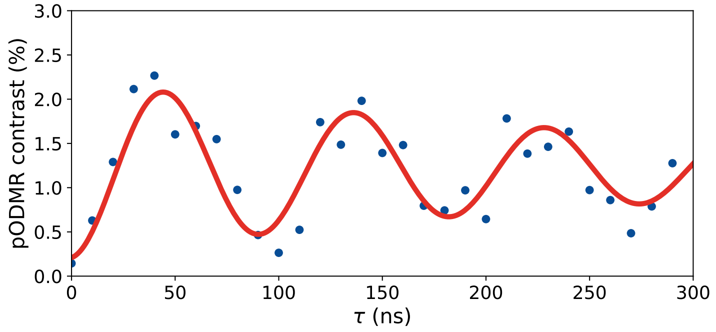

Supplementary Fig. 4. Rabi measurement for the defect presented in main text, Fig. 2, used to calibrate the duration of π pulses in subsequent experiments.

Supplementary Note 4. BACKGROUND CORRECTION OF INTENSITY AUTOCORRELATION MEASUREMENTS

We conduct intensity autocorrelation measurements using Hanbury-Brown Twiss interferometry. In these experiments, the fluorescence collection fibre is connected to a 50:50 fibre beamsplitter, with each end coupled into a single-photon avalanche photodiode. We include the effect of the background photoluminescence by renormalizing the $g^{(2)}(t)$ trace based on the parameter p such that

$$g_p^{(2)}(t) = \frac{g^{(2)}(t) - (1 - p^2)}{p^2} \tag{1}$$

where p is the fraction of total PL coming from the emitter [1]. We estimate p from confocal scans of the emitter.

Supplementary Note 5. KINETIC MODEL OF THE SYSTEM

We build a model with the goal of simulating the photoluminescence (PL) over time in various time-resolved and pulsed-microwave experiments. In order to do this, we build a set of rate equations describing the time-evolution of population of each level, and use this to simulate the PL

$$\dot{\boldsymbol{\rho}} = \mathbf{M}(P_{\text{opt}}, P_{\text{MW}})\boldsymbol{\rho} \tag{2}$$

where $\mathbf{M}$ is the matrix describing the transfer of population from level j into level i, and this is a function of the optical power P_{opt} and the microwave power P_{MW}. In the basis given by $\chi = \{\boldsymbol{\rho}_G, \boldsymbol{\rho}_E, \boldsymbol{\rho}_{S0}\}$, where $\boldsymbol{\rho}_G = \{\rho_{Gz}, \rho_{Gy}, \rho_{Gx}\}$ and $\boldsymbol{\rho}_E = \{\rho_{Ez}, \rho_{Ey}, \rho_{Ex}\}$, this can be written as

$$\begin{bmatrix} \dot{\boldsymbol{\rho}_G} \\ \dot{\boldsymbol{\rho}_E} \\ \dot{\boldsymbol{\rho}_{S0}} \end{bmatrix} = \begin{bmatrix} -\boldsymbol{\Gamma}_G + \boldsymbol{\Gamma}_{T_1} + \boldsymbol{\Omega}_{\text{MW}}(P_{\text{MW}}) & \boldsymbol{\Gamma}_{E\to G} & \mathbf{k}_{S0\to G} \\ \boldsymbol{\Gamma}_{G\to E}(P_{\text{opt}}) & -\boldsymbol{\Gamma}_E & \mathbf{0} \\ \mathbf{0} & \mathbf{k}_{E\to S0} & -\boldsymbol{\Gamma}_{S0} \end{bmatrix} \begin{bmatrix} \boldsymbol{\rho}_G \\ \boldsymbol{\rho}_E \\ \boldsymbol{\rho}_{S0} \end{bmatrix} \tag{3}$$

with

$$\begin{aligned} \boldsymbol{\Gamma}_{T_1} &= \gamma_{T_1} \begin{bmatrix} 0 & 1 & 1 \\ 1 & 0 & 1 \\ 1 & 1 & 0 \end{bmatrix} \\ \boldsymbol{\Omega}_{\text{MW}}(P_{\text{MW}}) &= \Omega_{\text{MW}}(P_{\text{MW}}) \begin{bmatrix} 0 & 1 & 0 \\ 1 & 0 & 0 \\ 0 & 0 & 0 \end{bmatrix} \\ \boldsymbol{\Gamma}_{E\to G} &= \Gamma_{E\to G} \begin{bmatrix} 1 & 0 & 0 \\ 0 & 1 & 0 \\ 0 & 0 & 1 \end{bmatrix} \end{aligned} \tag{4}$$

$$\mathbf{\Gamma}_{G\to E}(P_{\text{opt}}) = \Gamma_{G\to E}(P_{\text{opt}}) \begin{bmatrix} 1 & 0 & 0 \\ 0 & 1 & 0 \\ 0 & 0 & 1 \end{bmatrix}$$

$$\mathbf{k}_{S0\to G} = \begin{bmatrix} k_{S0\to Gz} \\ k_{S0\to Gy} \\ k_{S0\to Gx} \end{bmatrix}$$

$$\mathbf{k}_{E\to S0} = \begin{bmatrix} k_{Ez\to S0} & k_{Ey\to S0} & k_{Ex\to S0} \end{bmatrix}$$

$$\mathbf{\Gamma}_{G} = \begin{bmatrix} 2\gamma_{T_1} + \Gamma_{G\to E}(P_{\text{opt}}) + \Omega_{\text{MW}}(P_{\text{MW}}) & 0 & 0 \\ 0 & 2\gamma_{T_1} + \Gamma_{G\to E}(P_{\text{opt}}) + \Omega_{\text{MW}}(P_{\text{MW}}) & 0 \\ 0 & 0 & 2\gamma_{T_1} + \Gamma_{G\to E}(P_{\text{opt}}) \end{bmatrix}$$

$$\mathbf{\Gamma}_{E} = \begin{bmatrix} \Gamma_{E\to G} + k_{E0\to S0} & 0 & 0 \\ 0 & \Gamma_{E\to G} + k_{E+\to S0} & 0 \\ 0 & 0 & \Gamma_{E\to G} + k_{E-\to S0} \end{bmatrix}$$

$$\mathbf{\Gamma}_{S0} = \begin{bmatrix} k_{S0\to Gz} + k_{S0\to Gy} + k_{S0\to Gx} \end{bmatrix} \tag{5}$$

with rates defined in Fig. 2a of the main text.

We get the population as a function of time by solving this set of coupled differential equations. In order to do this in a computationally inexpensive way, we can rewrite

$$\mathbf{M} = \mathbf{U}_{P_{\text{opt}},P_{\text{MW}}} \boldsymbol{\lambda}_{P_{\text{opt}},P_{\text{MW}}} \mathbf{U}^{-1}_{P_{\text{opt}},P_{\text{MW}}} \tag{6}$$

where $\mathbf{U}_{P_{\text{opt}},P_{\text{MW}}}$ is the set of normalized eigenvectors of $\mathbf{M}(P_{\text{opt}}, P_{\text{MW}})$, and $\boldsymbol{\lambda}_{P_{\text{opt}},P_{\text{MW}}}$ is the diagonal matrix with eigenvalues of $\mathbf{M}(P_{\text{opt}}, P_{\text{MW}})$. Using this, we assume a solution to Eq. 2 of the form

$$\boldsymbol{\rho}(t) = \mathbf{U}_{P_{\text{opt}},P_{\text{MW}}} e^{(\boldsymbol{\lambda}_{P_{\text{opt}},P_{\text{MW}}} t)} \mathbf{U}^{-1}_{P_{\text{opt}},P_{\text{MW}}} \boldsymbol{\rho}(t_0) \tag{7}$$

In this way, the distribution of population over time is fully defined by the initial state $\boldsymbol{\rho}(t_0)$ and the set of rates included in $\mathbf{M}(P_{\mathrm{opt}}, P_{\mathrm{MW}})$. The eigenstate of $\mathbf{M}(P_{\mathrm{opt}}, P_{\mathrm{MW}})$ with eigenvalue $\lambda = 0$ gives the steady state population under a certain driving condition, $\boldsymbol{\rho}_{\mathrm{ss}}(P_{\mathrm{opt}}, P_{\mathrm{MW}})$. Finally, for a given population distribution, we assume that the photoluminescence is proportional to the radiative relaxation rate of each excited state sublevel times its population, such that

$$\mathrm{PL}(t) = \sum \boldsymbol{\Gamma}_{E\to G} \boldsymbol{\rho}_E(t) \tag{8}$$

We use this algorithm to calculate the population evolution – and resulting photoluminescence – of various experiments.

A. $g^{(2)}(t)$ experiments

To simulate the time-dependence observed in $g^{(2)}(t)$ experiments, we solve for

$$\boldsymbol{\rho}_{g^{(2)}}(t) = \mathbf{U}_{P_{\mathrm{opt}},0} e^{(\boldsymbol{\lambda}_{P_{\mathrm{opt}},0} t)} \mathbf{U}^{-1}_{P_{\mathrm{opt}},0} \boldsymbol{\rho}_{g^{(2)}}(0) \tag{9}$$

where $\boldsymbol{\rho}_{g^{(2)}}(0)$ is the initial state of the system immediately after it emits a photon. This initial state is given by [2]

$$\boldsymbol{\rho}_{g^{(2)}}(0) = \begin{bmatrix} \mathbf{0} & \frac{\boldsymbol{\Gamma}_{E\to G}}{\mathbf{e}^{\top}\boldsymbol{\Gamma}_{E\to G}\mathbf{e}} & \mathbf{0} \\ \mathbf{0} & \mathbf{0} & \mathbf{0} \\ \mathbf{0} & \mathbf{0} & \mathbf{0} \end{bmatrix} \begin{bmatrix} 0 \\ \frac{\boldsymbol{\rho}_{\mathrm{ss},E}(P_{\mathrm{opt}},0)}{\mathbf{e}^{\top}\boldsymbol{\rho}_{\mathrm{ss},E}(P_{\mathrm{opt}},0)} \\ 0 \end{bmatrix} \tag{10}$$

where $\mathbf{e}$ is a 3×1 vector with 1 at all entries. The probability of collecting a photon at a time t after the initial photon detection is determined by the system PL at that time. In a $g^{(2)}(t)$ experiment, we are measuring the probability of detecting a photon a time t after an initial photon emission, normalized by the unconditional probability of detecting a photon. Thus, the simulated $g^{(2)}(t)$ curve will be given by

$$g^{(2)}(t) = \frac{\sum \boldsymbol{\Gamma}_{E\to G} \boldsymbol{\rho}_{g^{(2)},E}(t)}{\sum \boldsymbol{\Gamma}_{E\to G} \boldsymbol{\rho}_{ss,E}(P_{\mathrm{opt}}, 0)} \tag{11}$$

where we have added a normalization factor corresponding to the probability of measuring

a photon if the system is in the steady state.

B. Spin-dependent initialisation experiment

In order to simulate the spin-dependent initialisation of the system (Fig. 2c of main text) we investigate the relative change in PL intensity a time $\tau_{\rm r}$ after the start of an optical pulse due to the presence of a microwave pulse. We thus study the time-evolution of the population in the presence of optical drive in a reference experiment (without MW drive) and in a signal experiment (with a MW pulse between two subsequent optical pulses).

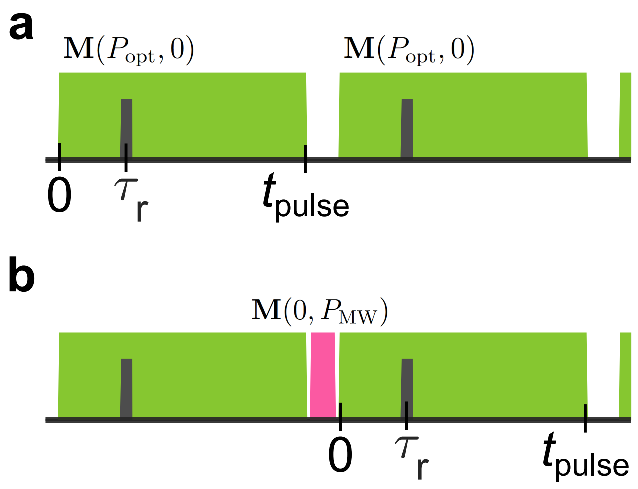

Supplementary Fig. 5. **Spin-dependent initialisation**. **a** Reference and **b** signal sequences. Green blocks represent optical pulses, pink block represents a microwave π pulse, and gray blocks represent a readout interval during which photons are collected. The readout interval is scanned across the duration of the optical pulse.

Supplemetary Fig. 5 shows the reference (a) and signal (b) pulse sequences, with relevant times specified. The optical pulse (green block) is chosen to be long (100s of microseconds) such that at $t_{\rm pulse}$ the system has settled into the steady state $\boldsymbol{\rho}_{\rm ss}(P_{\rm opt}, 0)$ given by the eigenstate of $\mathbf{M}(P_{\rm opt}, 0)$ with eigenvalue $\lambda = 0$. We assume that the optical drive is weak, such that at any time the population in the excited state is only a small fraction of the entire population. Since the time between the two optical pulses is small ($\sim$ 100 ns), we can assume that, for the reference experiment, the initial state $\boldsymbol{\rho}_{\rm ref}(0)$ is given by $\boldsymbol{\rho}_{\rm ss}(P_{\rm opt}, 0)$. In contrast, for the signal experiment we assume that the initial state is given by $\boldsymbol{\rho}_{\rm sig}(0) = \boldsymbol{\Pi}\boldsymbol{\rho}_{\rm ss}(P_{\rm opt}, 0)$, where $\boldsymbol{\Pi}$ is the operator that swaps ρ_{Gz} and ρ_{Gy}. Using these expressions for $\boldsymbol{\rho}_{\rm ref}(0)$ and $\boldsymbol{\rho}_{\rm sig}(0)$, we then calculate the population dependence on $\tau_{\rm r}$,

$$\boldsymbol{\rho}_{\mathrm{ref}}(\tau_{\mathrm{r}}) = \mathbf{U}_{P_{\mathrm{opt}},0} e^{(\boldsymbol{\lambda}_{P_{\mathrm{opt}},0}\tau_{\mathrm{r}})} \mathbf{U}^{-1}_{P_{\mathrm{opt}},0} \boldsymbol{\rho}_{\mathrm{ref}}(0)$$
$$\boldsymbol{\rho}_{\mathrm{sig}}(\tau_{\mathrm{r}}) = \mathbf{U}_{P_{\mathrm{opt}},0} e^{(\boldsymbol{\lambda}_{P_{\mathrm{opt}},0}\tau_{\mathrm{r}})} \mathbf{U}^{-1}_{P_{\mathrm{opt}},0} \boldsymbol{\rho}_{\mathrm{sig}}(0) \tag{12}$$

and combine this with Eq. 8 to obtain the contrast as a function of τ_{r}:

$$C_{\mathrm{PL}}(\tau_{\mathrm{r}}) = \frac{\sum(\boldsymbol{\Gamma}_{E\to G}\boldsymbol{\rho}_{\mathrm{sig},E}(\tau_{\mathrm{r}}) - \boldsymbol{\Gamma}_{E\to G}\boldsymbol{\rho}_{\mathrm{ref},E}(\tau_{\mathrm{r}}))}{\sum(\boldsymbol{\Gamma}_{E\to G}\boldsymbol{\rho}_{\mathrm{ref},E}(\tau_{\mathrm{r}}))} \tag{13}$$

C. Modified spin-relaxation experiment

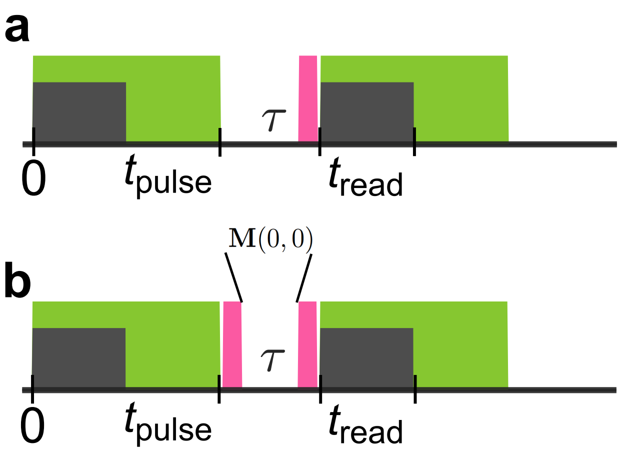

Supplementary Fig. 6. **Modified spin relaxation experiment**. **a** Reference and **b** signal sequences. Green blocks represent optical pulses, pink blocks represents a microwave π pulse, and gray blocks represent a readout interval during which photons are collected. The time interval between the two microwave pulses in the signal sequence, indicated by τ, is scanned during the experiment.

In order to simulate the behavior of the system in a modified spin-relaxation experiment (Fig. 2d of main text) we proceed similarly as above, but now investigate the dependence of a variable interval of length τ during the pulses. Supplementary Fig. 5 shows the reference (a) and signal (b) pulse sequences, with relevant times specified.

At $t = t_{\mathrm{pulse}}$, i.e. at the end of a long optical pulse, the system is in state $\boldsymbol{\rho}_{\mathrm{ss}}(P_{\mathrm{opt}}, 0)$. In the reference experiment, this is followed by a variable delay τ, where the population evolves as determined by the rate matrix $\mathbf{M}(0, 0)$. In the signal experiment, the time delay τ is preceded by a microwave pulse represented by the operator $\mathbf{\Pi}$. In both reference and signal experiments, a microwave pulse is applied after the delay τ and just before the arrival

of the optical pulse. The state of the system at $t = 0$, when the optical pulse arrives, is given by

$$\begin{aligned}\boldsymbol{\rho}_{\text{ref}}(0) &= \boldsymbol{\Pi}(\mathbf{U}_{0,0}e^{(\boldsymbol{\lambda}_{0,0}\tau)}\mathbf{U}_{0,0}^{-1})\boldsymbol{\rho}_{\text{ss}}(P_{\text{opt}},0)\\ \boldsymbol{\rho}_{\text{sig}}(0) &= \boldsymbol{\Pi}(\mathbf{U}_{0,0}e^{(\boldsymbol{\lambda}_{0,0}\tau)}\mathbf{U}_{0,0}^{-1})\boldsymbol{\Pi}\boldsymbol{\rho}_{\text{ss}}(P_{\text{opt}},0)\end{aligned} \tag{14}$$

The subsequent population evolution during the optical drive is then given by

$$\begin{aligned}\boldsymbol{\rho}_{\text{ref}}(t) &= \mathbf{U}_{P_{\text{opt}},0}e^{(\boldsymbol{\lambda}_{P_{\text{opt}},0}t)}\mathbf{U}_{P_{\text{opt}},0}^{-1}\boldsymbol{\rho}_{\text{ref}}(0)\\ \boldsymbol{\rho}_{\text{sig}}(t) &= \mathbf{U}_{P_{\text{opt}},0}e^{(\boldsymbol{\lambda}_{P_{\text{opt}},0}t)}\mathbf{U}_{P_{\text{opt}},0}^{-1}\boldsymbol{\rho}_{\text{sig}}(0)\end{aligned} \tag{15}$$

which we combine with Eq. 8 to calculate the PL during the optical drive pulse. We integrate this between $t = 0$ and $t = t_{\text{read}}$ to obtain the integrated PL during the readout time lasting approximately 100 μs:

$$\begin{aligned}C_{T_1}(\tau) &= \frac{\text{PL}_{\text{sig}} - \text{PL}_{\text{ref}}}{\text{PL}_{\text{ref}}},\\ \text{PL}_{\text{ref}} &= \int_0^{t_{\text{read}}} \sum \boldsymbol{\Gamma}_{E\to G}\boldsymbol{\rho}_{\text{ref}}(t)\,dt\\ \text{PL}_{\text{sig}} &= \int_0^{t_{\text{read}}} \sum \boldsymbol{\Gamma}_{E\to G}\boldsymbol{\rho}_{\text{sig}}(t)\,dt\end{aligned} \tag{16}$$

D. cwODMR contrast

In order to calculate the cwODMR contrast, we compare the steady-state PL in the presence of simultaneous optical and microwave drives ($\boldsymbol{\rho}_{\text{ss}}(P_{\text{opt}}, P_{\text{MW}})$) to the steady-state PL in the presence of optical drive and absence of microwave drive ($\boldsymbol{\rho}_{\text{ss}}(P_{\text{opt}}, 0)$). These are respectively determined by the eigenstates of $\mathbf{M}(P_{\text{opt}}, P_{\text{MW}})$ and $\mathbf{M}(P_{\text{opt}}, P_{\text{MW}} = 0)$ with eigenvalues equal to zero. This gives, for the cwODMR contrast,

$$C_{\text{cw}} = \frac{\sum(\boldsymbol{\Gamma}_{E\to G}\boldsymbol{\rho}_{\text{ss},E}(P_{\text{opt}}, P_{\text{MW}}) - \boldsymbol{\Gamma}_{E\to G}\boldsymbol{\rho}_{\text{ss},E}(P_{\text{opt}}, 0))}{\sum \boldsymbol{\Gamma}_{E\to G}\boldsymbol{\rho}_{\text{ss},E}(P_{\text{opt}}, 0)} \tag{17}$$

E. Fitting Procedure

In order to determine the parameters presented in the schematics of Fig. 2a of the main text for our system, we fit the predictions of the model presented in the section above to the experimental results presented in Fig. 2b-d of the main text. We do this by minimizing the error given by

$$\delta_{\text{total}} = \delta_{g^{(2)}} + \delta_{\text{PL}} + \delta_{T_1} \tag{18}$$

where

$$\begin{aligned}
\delta_{g^{(2)}} &= \sum_{t_i} \tfrac{1}{N}(g^{(2)}_{\text{calc}}(t_i) - g^{(2)}_{\text{exp}}(t_i))^2, \\
\delta_{\text{PL}} &= \sum_{\tau_{\text{r},i}} \tfrac{1}{N}(A_{\text{PL}} C_{\text{PL,calc}}(\tau_{\text{r},i}) - C_{\text{PL,exp}}(\tau_{\text{r},i}))^2, \\
\delta_{T_1} &= \sum_{\tau_i} \tfrac{1}{N}(A_{T_1} C_{T_1,\text{calc}}(\tau_i) - C_{T_1,\text{exp}}(\tau_i))^2,
\end{aligned} \tag{19}$$

with $g^{(2)}_{\text{calc}}(t)$, $C_{\text{PL,calc}}(t)$ and $C_{T_1,\text{calc}}(t)$ defined in Eqs. 11, 13 and 16, respectively. The subindices calc and exp refer respectively to calculated or experimentally observed quantities, and the $\frac{1}{N}$ factor normalizes for the number of observations made in each measurement. The prefactors A_{PL} and A_{T_1} are phenomenological scaling factors between 0 and 1 that account for the fact that we often observe lower contrast in pulsed than cw microwave experiments, a feature that could arise from imperfect microwave delivery due to impedance mismatches in our microwave line. Finally, we constrain the fit to parameter combinations that provide calculated cwODMR contrast at saturated microwave drive condition equal to or higher to the measured cwODMR contrast, and calculated PL equal or higher than the experimental PL.

We note that, as a starting point, we do not know the order of magnitude of several of the rates in the model. In order to accurately sample rates across different orders of magnitudes, we reparametrize the model in terms of the parameters $\{\alpha_{E\to G}, \alpha_{G\to E}, \alpha_{S0\to G}, \alpha_{E\to S0}, \alpha_{T_1}, k_G, k_E, A_{\text{PL}}, A_{T_1}\}$, such that

$$
\begin{aligned}
\alpha_{E\to G} &= \log\left(\Gamma_{E\to G}\right)\\
\alpha_{G\to E} &= \log\left(\Gamma_{G\to E}\right)\\
\alpha_{S0\to G} &= \log\left(k_{S0\to Gx} + k_{S0\to Gy} + k_{S0\to Gz}\right)\\
\alpha_{E\to S0} &= \log\left(k_{Ex\to S0} + k_{Ey\to S0} + k_{Ez\to S0}\right)\\
\alpha_{T_1} &= \log\left(\gamma_{T_1}\right)\\
k_G &= (k_{S0\to Gx} + k_{S0\to Gz})/k_{S0\to Gy}\\
k_E &= (k_{Ex\to S0} + k_{Ez\to S0})/k_{Ey\to S0}
\end{aligned}
\tag{20}
$$

and fit for these parameters instead. We obtain the best estimates for the parameters $\{\Gamma_{E\to G}, \Gamma_{G\to E}, k_{Ex,z\to S0}, k_{Ey\to S0}, k_{S0\to Gx,z}, k_{S0\to Gy}, \gamma_{T_1}, A_{\mathrm{PL}}, A_{T_1}\}$ from minimizing the error as described above. We assume here that $k_{Ex,z\to S0} = k_{Ex\to S0} = k_{Ez\to S0}$, and analogously for the reverse intersystem crossing rates in order to minimize the number of fit parameters involved.

F. Uncertainties in Estimated Parameters

We estimate the errors in the extracted parameters through the following procedure:

1. We randomly sample a large number of combinations of parameters $\{\alpha_{E\to G}, \alpha_{G\to E}, \alpha_{S0\to G}, \alpha_{E\to S0}, \alpha_{T_1}, k_G, k_E, A_{\mathrm{PL}}, A_{T_1}\}$ within reasonable bounds, and calculate $\delta_{\mathrm{total}}, \delta_{g^{(2)}}, \delta_{\mathrm{PL}}, \delta_{T_1}$ for each combination of parameters.

2. We analyze whether the combination of parameters satisfies the hard bounds set by our experiment (*i.e.* calculated cwODMR $\geq$ experimental saturated cwODMR contrast, and calculated PL $\geq$ experimental PL) and discard instances where bounds are not met.

3. We remove combinations of parameters that give errors above 3 times the error of the best fit for each experiment

4. We histogram each parameter to obtain a probability distribution for each parameter that gives errors within 3 time the best-fit errors.

The result of this analysis is given in Supplementary Fig. 7, where occurrences are size and colour-coded: larger, blue circles represent smaller error while smaller, yellow circles represent configurations where errors are larger. The green vertical lines on Supplementary Fig. 7 represent the optical rates of best fit, which are presented in Tab. 1 in the main text. In some cases, the best-fit values of the parameters do not coincide with the peak of the probability density (red curves in Supplementary Fig. 7) for the parameters. We extract the error values for each parameter from the regions containing 99% of the area under the probability density functions (indicated by the vertical gray curves in Supplementary Fig. 7). We present these errors in Tab. 1 of the main text. The parameter that shows the highest uncertainty is the reverse intersystem crossing rate (from S0 to GS), with low-error occurrences spanning several orders of magnitude and peak-value of the probability density occurring close to the best-fit value reported by us originally in the manuscript. This indicates that the absolute value of the total reverse ISC rate is not bound by our model, and this rate has little influence on the experimental data. Despite this, the value of k_G, which sets the spin asymmetry in the reverse ISC process, is properly bound by our model and limited to 0.26 for this defect, indicating strong spin-selectivity in the reverse ISC. Certain combinations of rates do improve the fit of the model to the early spin-relaxation data; however, at the same time, these combinations incur in a poorer fit to the long-time delays in the intensity autocorrelation data. One possible explanation for this trade-off may be that our kinetic model is an oversimplification of the level structure for this defect. It is possible that other, higher lying excited states, in either the singlet or triplet manifolds, are involved in the optical cycle and influence the photoluminescence traces we measure.

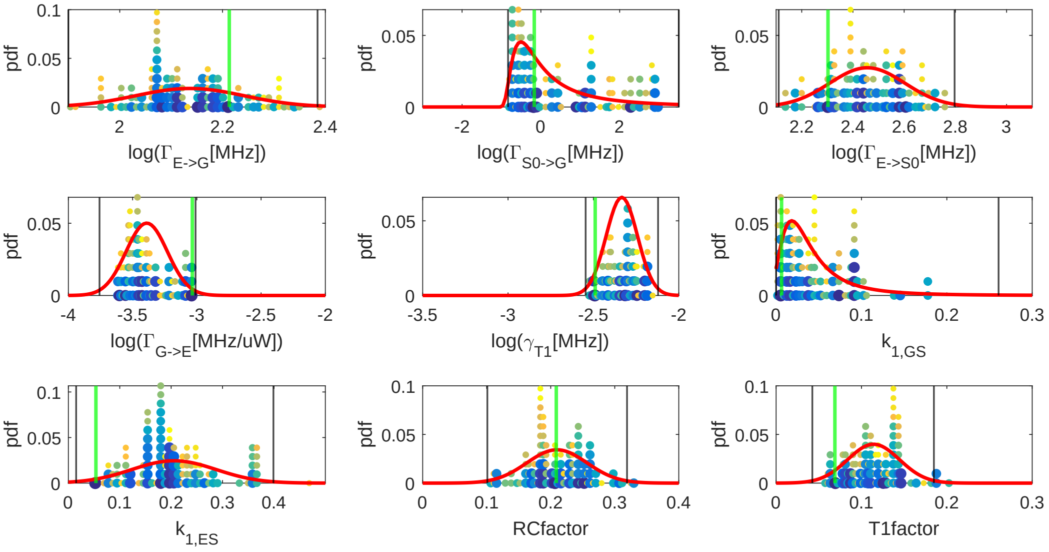

Supplementary Fig. 7. **Probability density functions of each of the 9 parameters involved in the fitting procedure**, obtained by investigating >1x10^6 samples of randomly obtained combinations of parameters, and calculating both overall and experiment-specific errors. Here, we histogram the parameter values that give both overall and experiment-specific errors below 3x the error of the best fit. The circles corresponding to each sample are colour and size coded: samples that give smaller errors are given by large blue circles, whereas samples that give larger error are given by small yellow circles. The green vertical lines represent the parameter values of the best-fit to the data. Red curves are guide to the eye representing a probability density function obtained from the histograms. Gray vertical lines indicate the regions containing 99% of the area under the curve of the pdf.

Supplementary Note 6. COMPARISON BETWEEN MODELS WITH *S=1* IN GROUND STATE OR METASTABLE STATE

We consider a model consisting of spin singlet ground and excited states, and a triplet metastable state, reminiscent of what is seen in the case of organic molecules like pentacene and the ST1 and TR12 defects in diamond [3–5]. We consider a model as presented in Supplementary Fig. 8, and proceed to obtain a global fit of this model to the experimental results. The results of this fit are presented in Supplementary Fig. 8. While the model is able to capture the behaviour observed in the intensity autocorrelation and the spin-dependent initialisation experiments, it is not able to capture the behavior of the modified T_1 experiment.

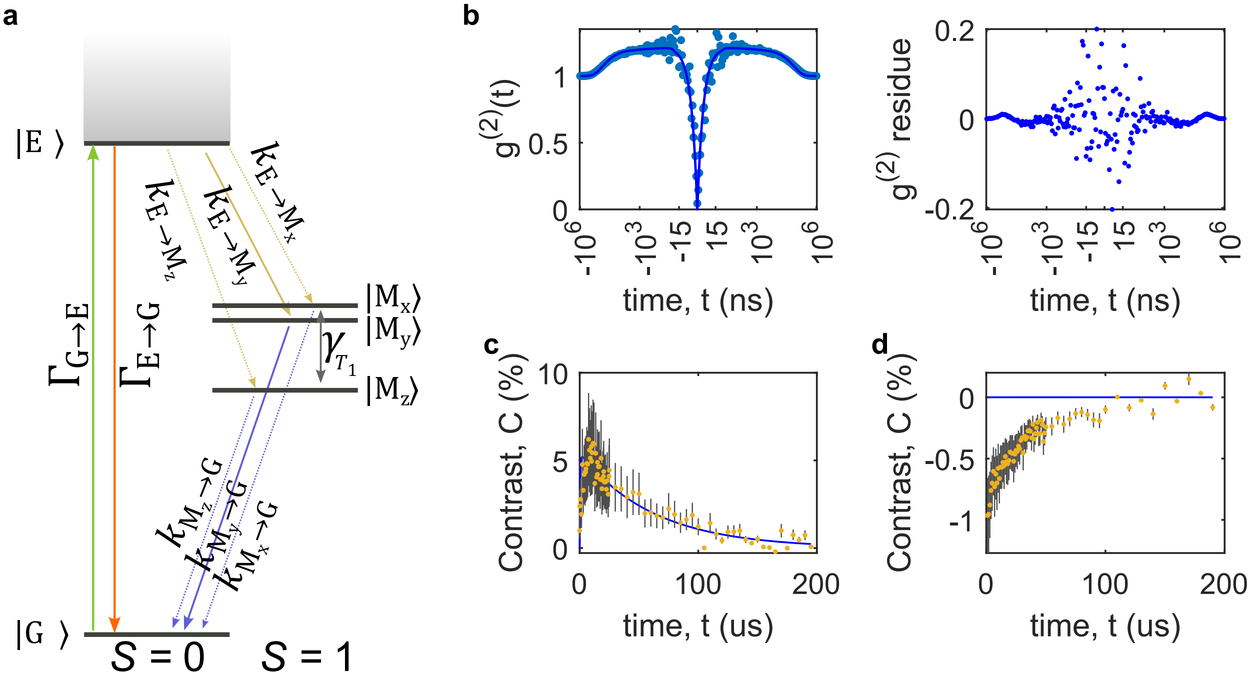

Supplementary Fig. 8. **Model with a spin-triplet in the metastable state, and spin singlet ground and optically excited states.** **a** Description of the model and relevant rates; **b** background-corrected results of an intensity autocorrelation experiment (blue circles), with fit result (blue curve) and residuals of the fit (right panel). **c** Experimental results (yellow circles) of the spin-dependent initialisation experiment and **d** modified T_1 experiment, accompanied by results of a fit of the model to the experimental data (blue curves).

In addition, we measure the spin-relaxation of different defects using two different pulse sequences as indicated in Supplementary Fig. 9. These sequences correspond to two different experiments probing the spin-dependent relaxation, where the contrast-inducing microwave

π pulse on resonance with a microwave resonance of the system occurs after (Seq. I) or before (Seq. II) a variable time τ. For a system with a spin-triplet in the metastable state, the spin-dependent pulsed-ODMR (pODMR) contrast decay in these experiments is expected to reflect the differences in spin-dependent reverse intersystem crossing rates [3]. In this case, we expect to observe relaxation times that differ significantly depending on whether the π pulse occurs before or after a wait time. In contrast, in the case of a ground-state spin triplet, the relaxation dynamics from either spin sublevel is expected to be similar and dominated by spin-lattice relaxation.

We perform this experiment on two different emitters respectively at room temperature and at 4 K, with results presented in Supplementary Fig. 10. We observe that the two sequences result in equal relaxation timescales for the decay of pODMR contrast. These results further support our assignment for the configuration of energy levels for this defect type, with a ground-state spin triplet and a metastable state spin singlet.

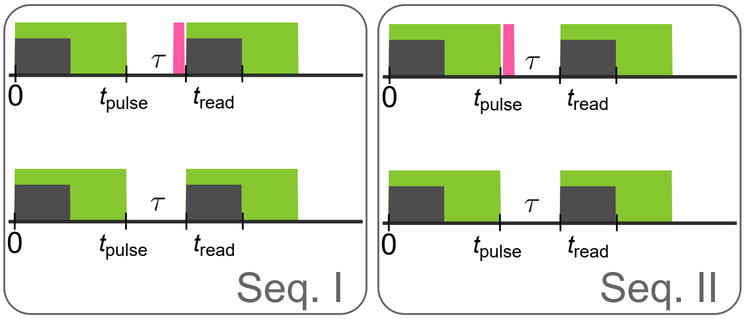

Supplementary Fig. 9. Pulse sequences used to measure the spin-dependent relaxation time. Green blocks represent optical pulses, pink blocks represents a microwave π pulse, and gray blocks represent a readout interval during which photons are collected. Sequences on the top indicate signal sequence, whereas the sequences in the bottom indicate the reference sequences. The time interval before (Seq. I) and after (Seq. II) the microwave pulse in the signal sequence, indicated by τ, is scanned during the experiment.

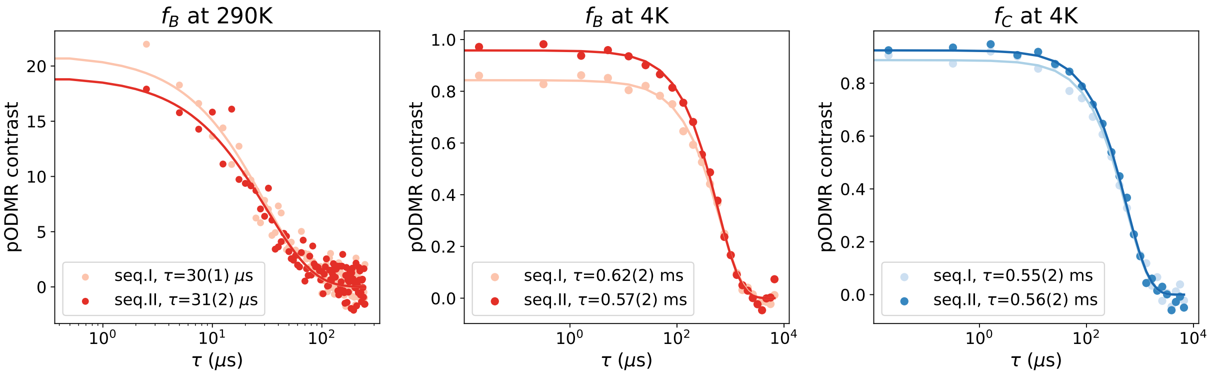

Supplementary Fig. 10. **Results of a spin-relaxation experiment** where the contrast-inducing microwave π pulse occurs after (Seq. I) or before (Seq. II) a variable delay time τ. Left panel presents the result of this experiment on a defect at room-temperature, and when the π pulse is on resonance with f_{B}. The middle and right panels present the result of this experiment on a defect at 4 K, and with the π pulse on resonance with f_{B} and f_{C}, respectively.

Supplementary Note 7. PODMR AND $g^2(t)$ DATA WITH ASSOCIATED GLOBAL FITS FOR ADDITIONAL DEFECTS

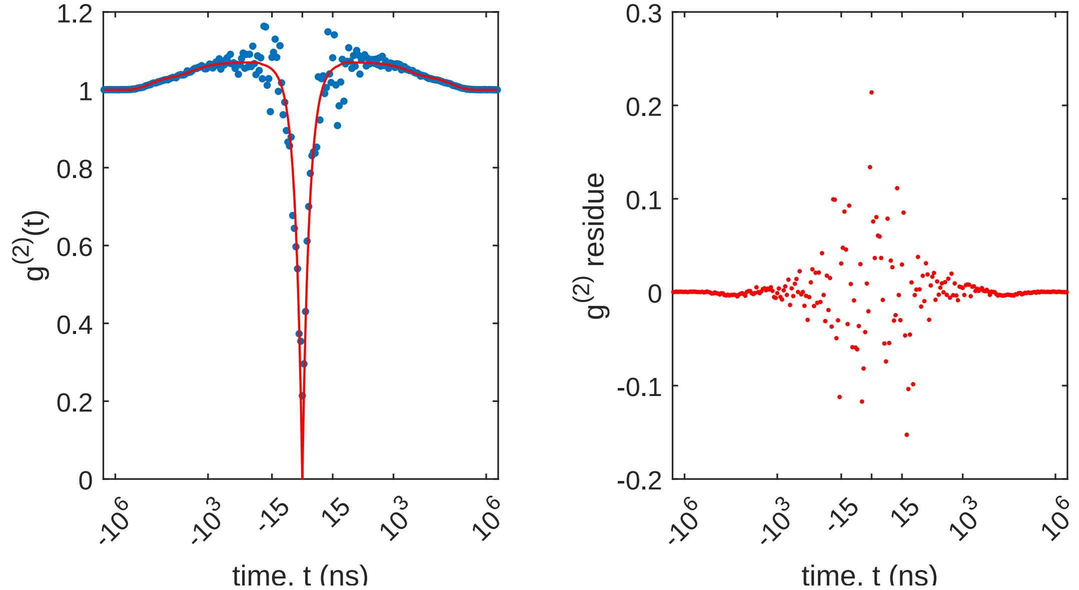

Supplementary Fig. 11. **Defect 4.** Background-corrected intensity autocorrelation used to extract rates in Supplementary Tab. I (left) and residual of the fit (right). Background is typically not more than 10% of emitter PL. Data is presented as blue circles, and result of the fit is presented as red curve.

Supplementary Tab. I. **Model parameters.** Summary of key parameters obtained from fitting the data in Figs. 11-15 to the model with a triplet ground state (main text, Fig. 2a).

Rate	$\Gamma_{\mathrm{G}\to\mathrm{E}}$	$\Gamma_{\mathrm{E}\to\mathrm{G}}$	$k_{\mathrm{E_x}\to S0}$	$k_{\mathrm{E_y}\to S0}$	$k_{\mathrm{E_z}\to S0}$	$k_{S0\to\mathrm{G_x}}$	$k_{S0\to\mathrm{G_y}}$	$k_{S0\to\mathrm{G_z}}$	γ_{T_1}	f_{B} Contrast
Unit	kHz/μW	MHz	MHz	MHz	MHz	kHz	kHz	kHz	kHz	%
Def4	0.18	118	114	220	114	26	248	26	1.0	3.7
Def3744	0.07	220	149	456	149	633	2661	633	8.3	4
Def15	0.9	143	34	388	34	5	856	5	0.3	9.3
Def3981	0.92	138	10	253	10	166	5200	166	0.23	9.5
DefJ19	0.92	163	5.4	190	5.4	2	675	2	3.2	12
Def12	7.1	168	21	529	21	35	2605	35	1.3	30

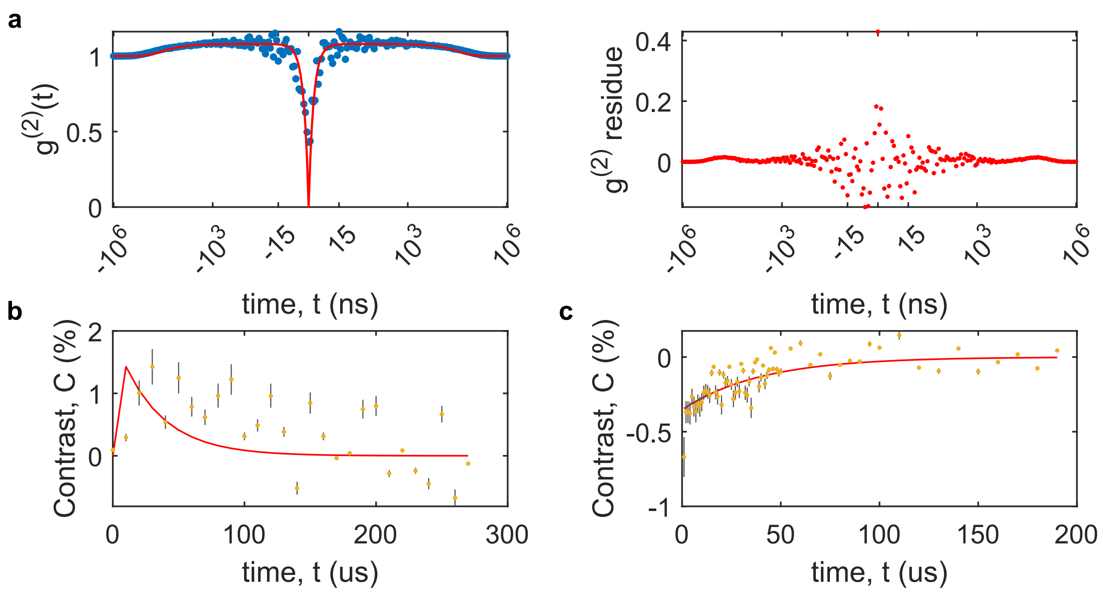

Supplementary Fig. 12. **Defect 3744.** Experimental data (blue and yellow circles) and results of fits (red curves) used to extract rates in Supplementary Tab. I. **a** Background-corrected intensity autocorrelation (left) and residual of the fit of the intensity autocorrelation experiment (right). Background is typically not more than 10% of emitter PL. **b** Spin-dependent initialisation, and **c** modified T_1. The error bars correspond to one standard deviation of the measured data.

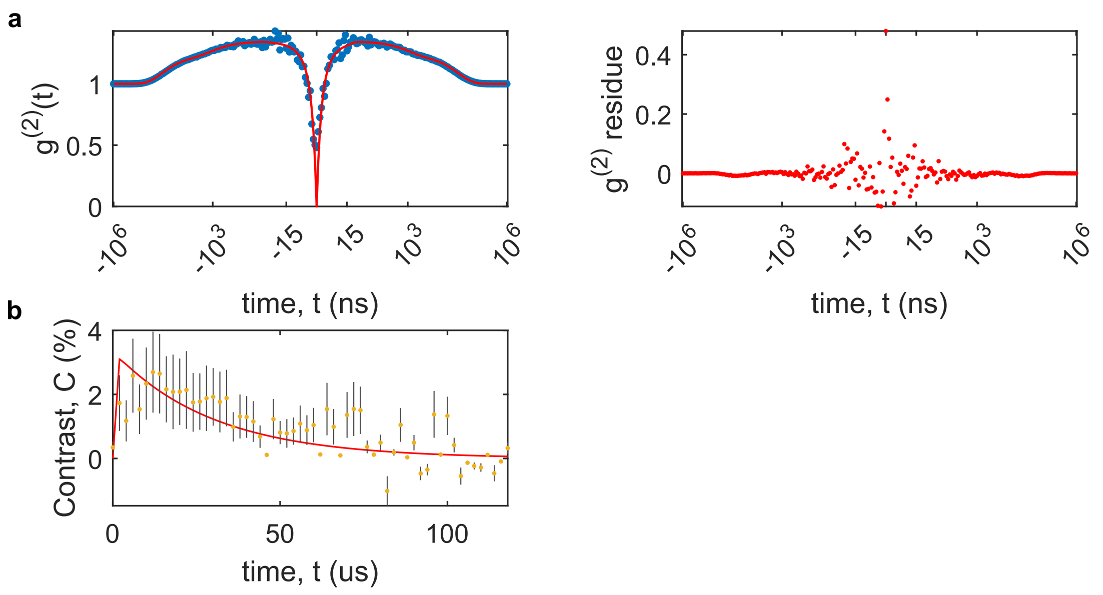

Supplementary Fig. 13. **Defect 15.** Experimental data (blue and yellow circles) and results of fits (red curves) used to extract rates in Supplementary Tab. I. **a** Background-corrected intensity autocorrelation (left) and residual of the fit of the intensity autocorrelation experiment (right). Background is typically not more than 10% of emitter PL. **b** Spin-dependent initialisation. The error bars correspond to one standard deviation of the measured data.

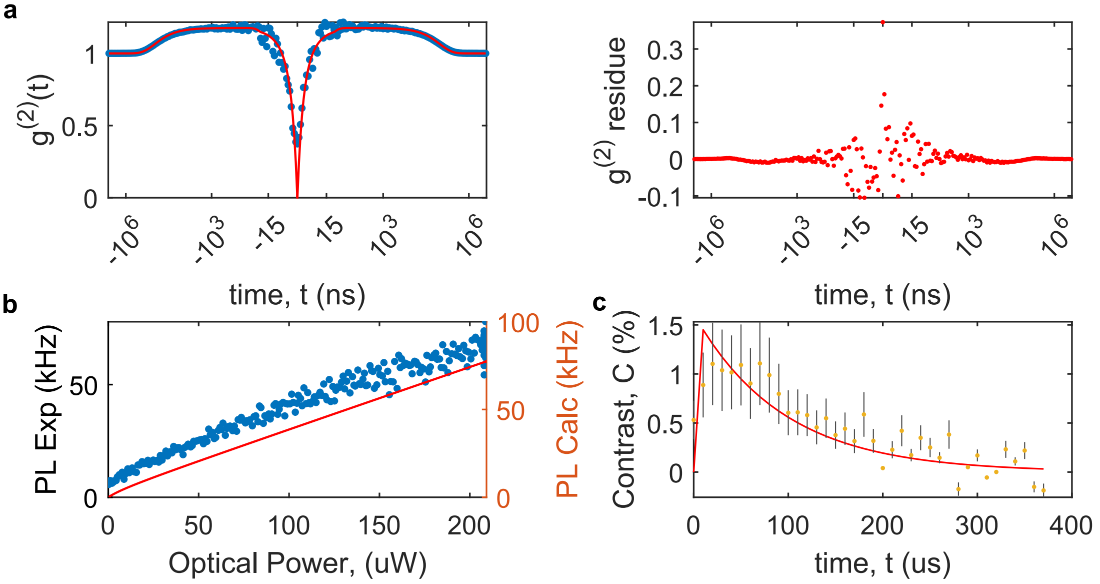

Supplementary Fig. 14. **Defect 3981.** Experimental data (blue and yellow circles) and results of fits (red curves) used to extract rates in Supplementary Tab. I. **a** Background-corrected intensity autocorrelation (left) and residual of the fit of the intensity autocorrelation experiment (right). Background is typically not more than 10% of emitter PL. **b** PL saturation and **c** Spin-dependent initialisation. The error bars correspond to one standard deviation of the measured data.

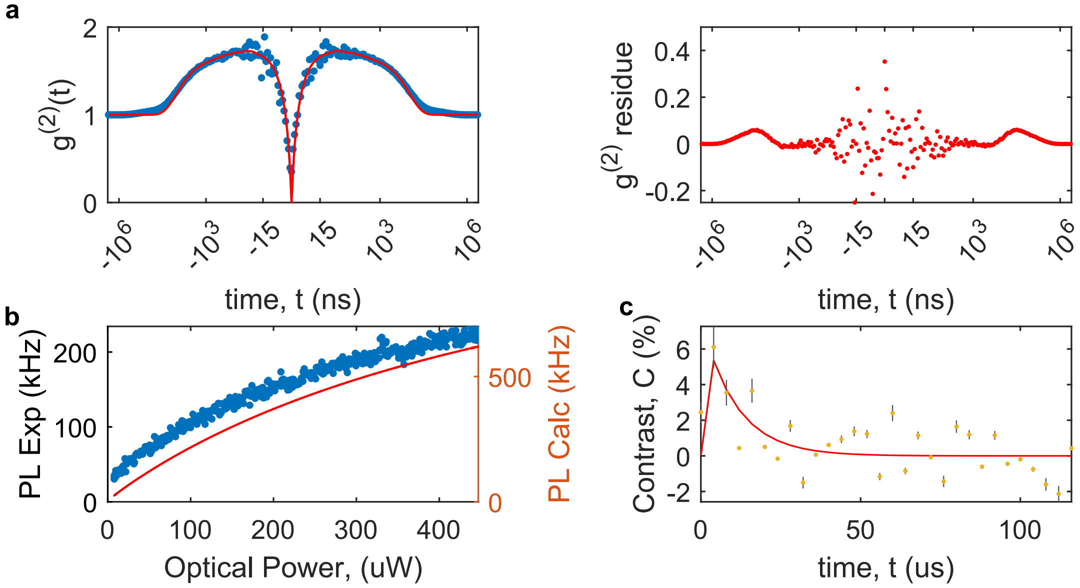

Supplementary Fig. 15. **Defect 12.** Experimental data (blue and yellow circles) and results of fits (red curves) used to extract rates in Supplementary Tab. I. **a** Background-corrected intensity autocorrelation (left) and residual of the fit of the intensity autocorrelation experiment (right). Background is typically not more than 10% of emitter PL. **b** PL saturation and **c** Spin-dependent initialisation. The error bars correspond to one standard deviation of the measured data.

Supplementary Note 8. CWODMR SPECTRA

A. cwODMR spectra associated with Figure 2e

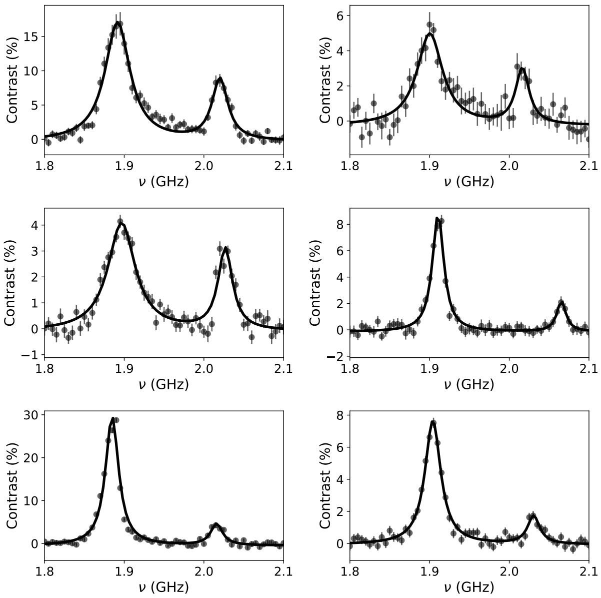

Supplementary Fig. 16. **cwODMR spectra associated with Figure 2e.** cwODMR Spectra taken under saturation conditions for different defects in the absence of magnetic field. The lower frequency peak corresponds to $f_{\rm B}$, the higher frequency peak to $f_{\rm C}$. The saturated contrast of each $f_{\rm B}$ transition is plotted in Fig. 2e. The error bars correspond to the standard error of the mean of the measured data.

B. cwODMR spectra associated with Figure 3a

CW ODMR spectra under 50 mT field are presented below, with $\phi = 90$, varying θ. This corresponds to rotation in the yz plane of the defect, such that $\theta = 0, \phi = 90$ is along z and $\theta = 90, \phi = 90$ is along y. Physical setup constraints restrict measurements at angles outside of the range presented. The left column (green) corresponds to the $f_{\rm A}$ transition, the middle column (red) to the $f_{\rm B}$ transition, and the right column (blue) to the $f_{\rm C}$ transition.

A Lorentzian fit to each spectrum is shown as a black curve, identifying the transition frequency for each resonance. In some cases, additional peaks are observed near $f_{\rm A}$, which we assign to replicas of the expected peaks, as they match the half-frequency transitions of $f_{\rm A}$ and $f_{\rm B}$. Their presence is due to the generation of second and third-order harmonics of

the microwave carrier frequency at high output powers (1.5 mW into amplifier, 1.6 W after amplification). The replicas disappear at lower microwave powers (<0.1 W after amplification).

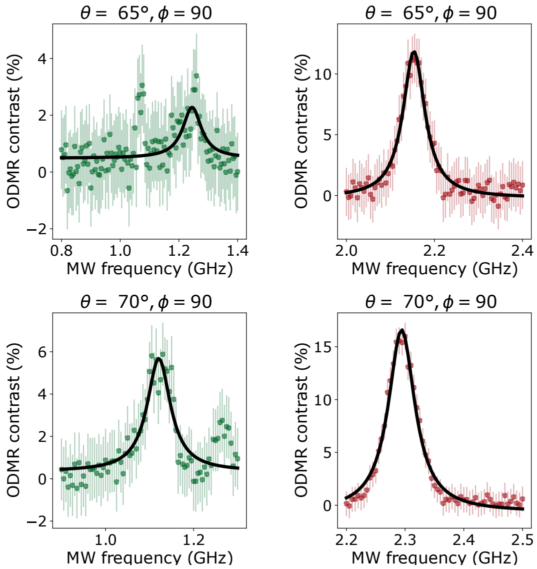

Supplementary Fig. 17. cwODMR spectra associated with Figure 3a at 50mT, with $\phi = 90$, varying θ. The error bars correspond to the standard error of the mean of the measured data.

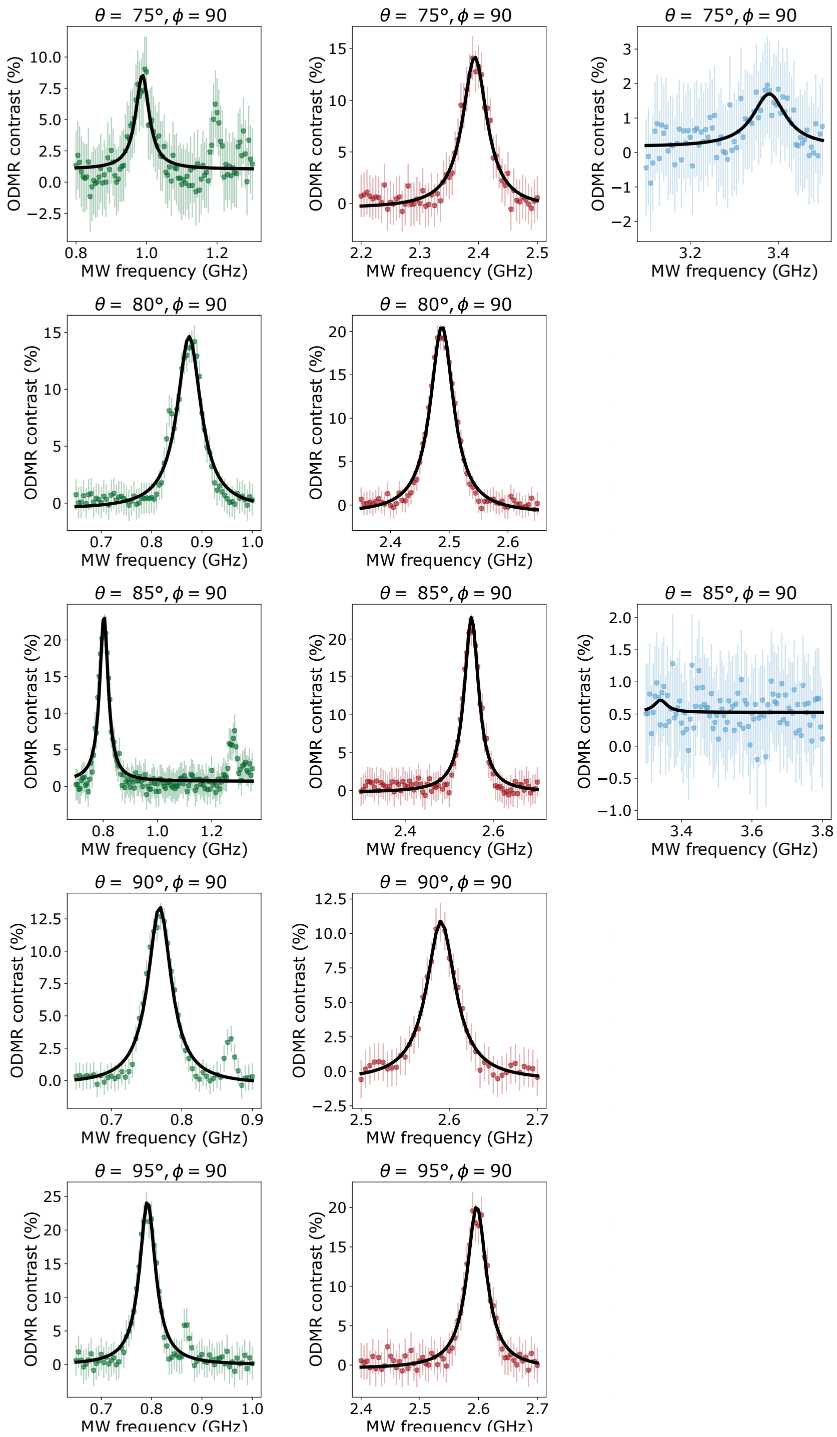

Supplementary Fig. 18. cwODMR spectra associated with Figure 3a at 50mT, with $\phi = 90$, varying θ. The error bars correspond to the standard error of the mean of the measured data.

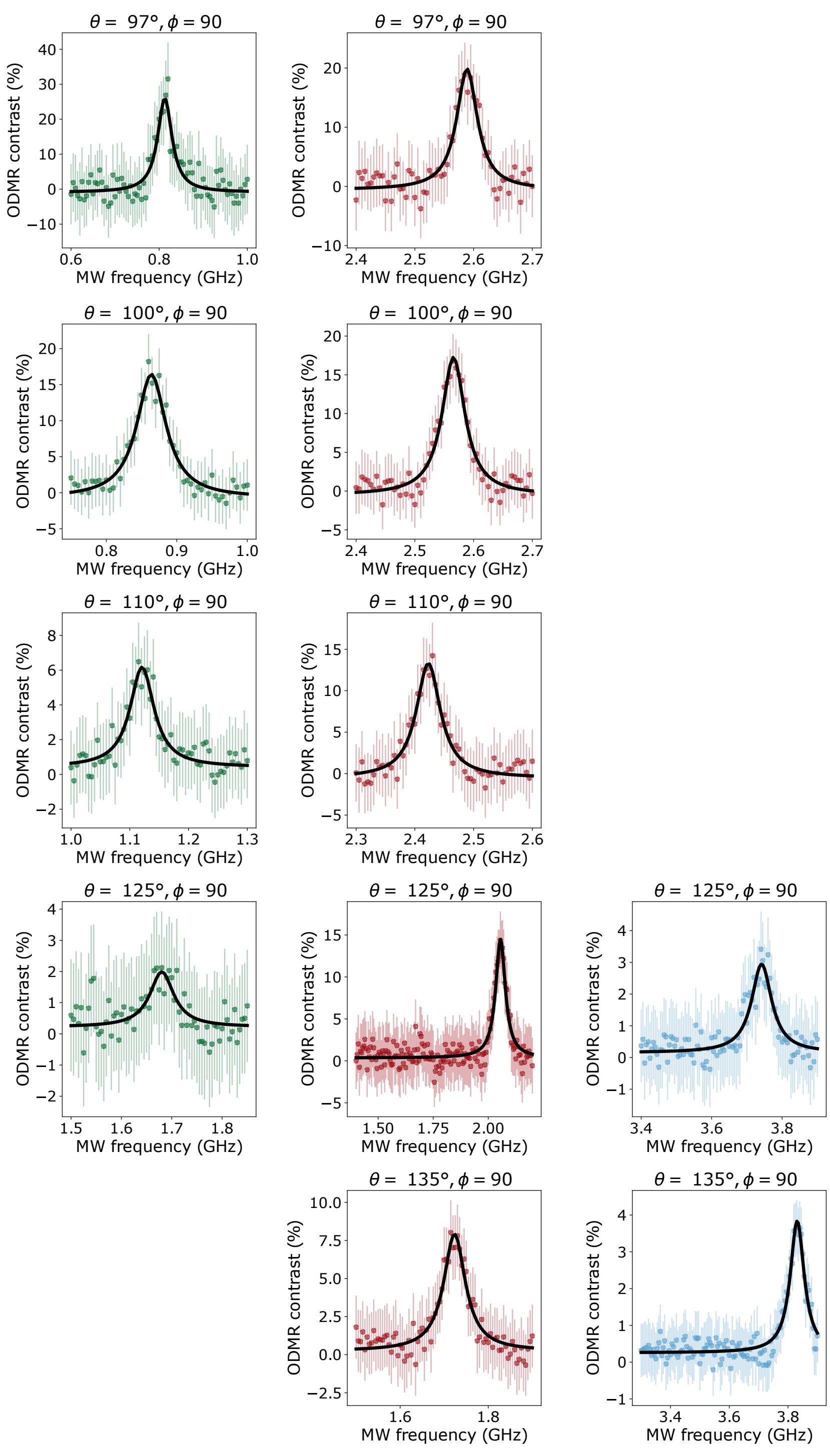

Supplementary Fig. 19. cwODMR spectra associated with Figure 3a at 50mT, with $\phi = 90$, varying θ.

C. cwODMR spectra associated with Figure 3c

CW ODMR spectra under 50 mT field are presented below, with varying ϕ, $\theta = 95$. This corresponds to rotation near the xy plane of the defect, where the x axis is given by $\phi = 0, \theta = 90$, and y is along $\phi = 90, \theta = 90$. A Lorentzian fit to each spectrum is shown as a black curve, identifying the transition frequency for each resonance. As before, we observe replicas alongside expected peaks in the spectra. Physical setup constraints restrict measurements at angles outside of the range presented. The left column (green) corresponds to the f_A transition and the right column (red) to the f_B transition.The contrast of f_C is not observable in this magnetic field orientation.

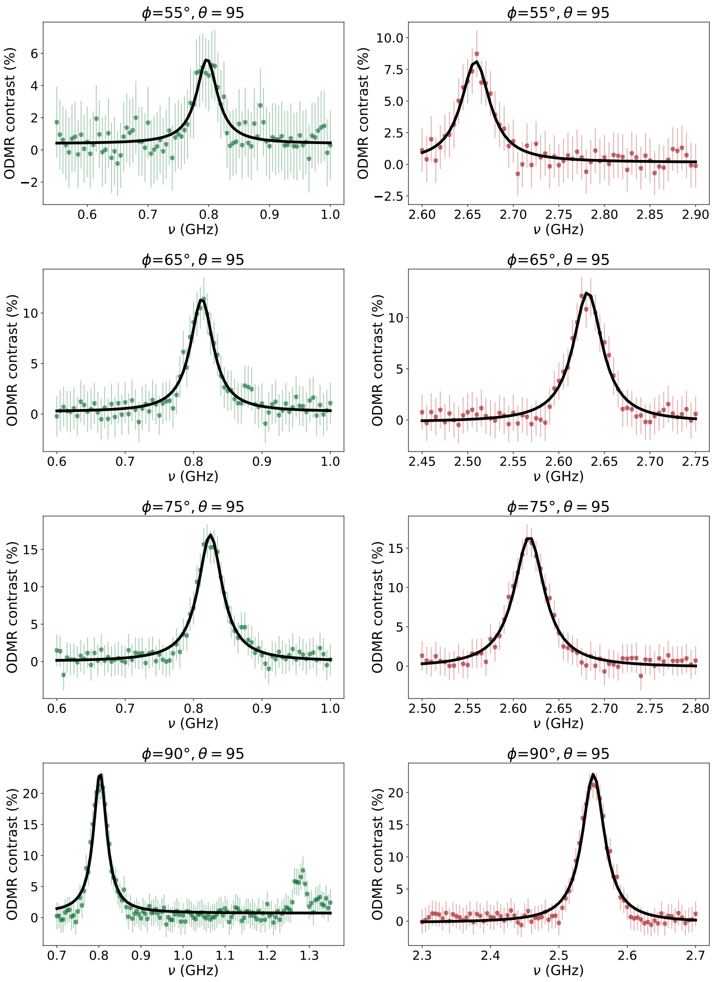

Supplementary Fig. 20. cwODMR spectra associated with Figure 3c at 50mT, with $\theta = 95$, varying ϕ.

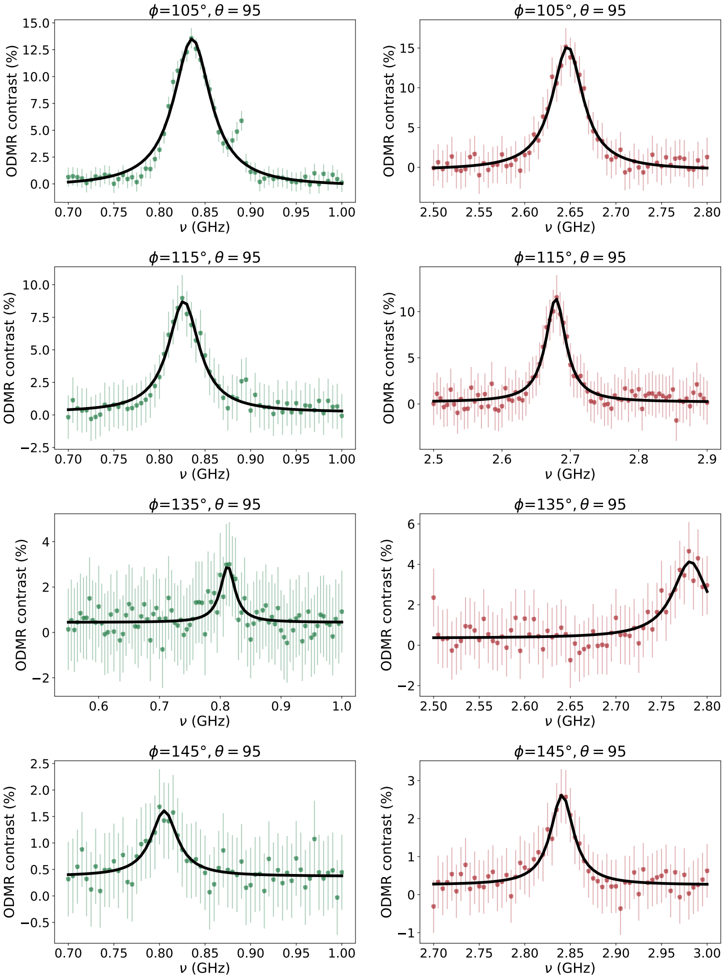

Supplementary Fig. 21. cwODMR spectra associated with Figure 3c at 50mT, with $\theta = 95$, varying ϕ.

Supplementary Note 9. EXCITED STATE ZERO-FIELD SPLITTING PARAMETERS

The excited-state zero-field splitting parameters, D_{ES}, E_{ES} directly govern the effect of bias magnetic field in mixing the excited-state zero-field eigenstates, and therefore have direct influence on the spin-selectivity of the direct intersystem crossing rates. Thus, we would expect that the magnitude of D_{ES}, E_{ES} would influence the spin-initialisation cycle at applied magnetic field.

In our experiments, we do not see spectroscopic signatures of spin transitions in the excited state that would allow us to extract the excited-state zero-field splitting parameters, D_{ES}, E_{ES}. In the absence of experimental values, we perform the calculations presented in the main text with the assumption $D_{\mathrm{ES}} = D_{\mathrm{GS}}$, $E_{\mathrm{ES}} = E_{\mathrm{GS}}$. Supplementary Fig. 22 shows that this assumption has little impact for the qualitative findings we present. In this figure, we present the sensitivity of each cwODMR resonance, with colour coding as in the main text, calculated for different values of D_{ES}, E_{ES}. For $D_{\mathrm{ES}} \neq D_{\mathrm{GS}}$, $E_{\mathrm{ES}} \neq E_{\mathrm{GS}}$, we observe some changes with the occurrence of *blind arcs* where no resonance presents significant contrast when $D_{\mathrm{ES}} \ll D_{\mathrm{GS}}$, $E_{\mathrm{ES}} \ll E_{\mathrm{GS}}$. Nonetheless, for most bias-field configurations, there is at least one cwODMR resonance that provides significant sensitivity.

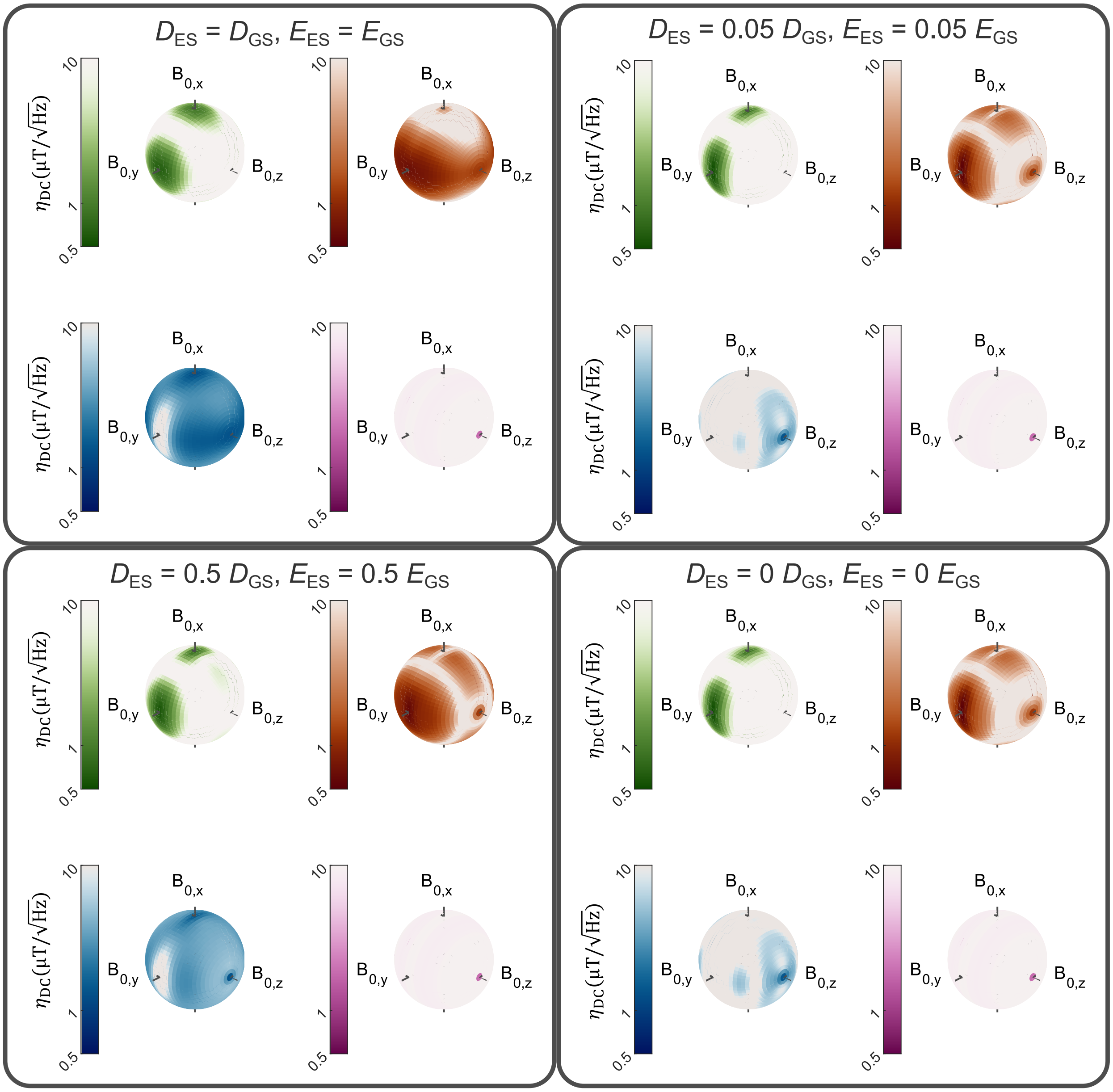

Supplementary Fig. 22. Calculated sensitivity of each cwODMR resonance plotted on a sphere, where the position on the sphere corresponds to the orientation of the 50-mT bias field, calculated for various excited-state zero-field splitting parameters.

Supplementary Note 10. SENSITIVITY RANGE

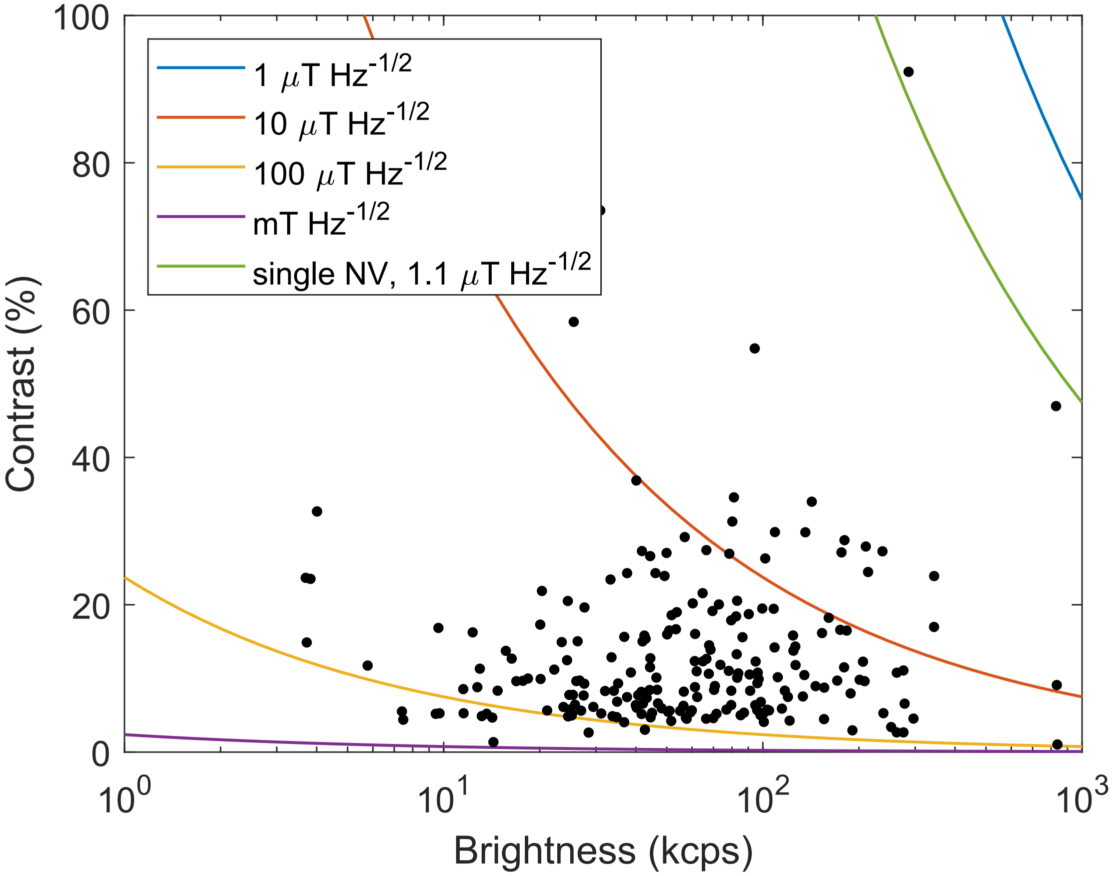

Supplementary Fig. 23. Contrast and brightness of individual defects measured in our setup. Contrast values observed in our setup range from 1 to 90%, whereas brightness values range from 4 kcps to ∼800 kcps. The curves indicate isothermals of sensitivity, as indicated in the legend, for defects with linewidth of 30 MHz. The green curve indicates the isothermal for which the hBN defects reported here have the same sensitivity as a typical shallow NV centre.

[1] R. E. K. Fishman, R. N. Patel, D. A. Hopper, T.-Y. Huang, and L. C. Bassett, "Photon-emission-correlation spectroscopy as an analytical tool for solid-state quantum defects," PRX Quantum **4**, 010202 (2023).

[2] A. L. Exarhos, D. A. Hopper, R. N. Patel, M. W. Doherty, and L. C. Bassett, "Magnetic-field-dependent quantum emission in hexagonal boron nitride at room temperature," Nat. Commun. **10**, 222 (2019).

[3] A. Mena, S. K. Mann, Cowley-Semple A., E. Bryan, S. Heutz, D. R. McCamey, M. Attwood, and S. L. Bayliss, "Room-temperature optically detected coherent control of molecular spins," Phys. Rev. Lett. **133**, 120801 (2024).

[4] P Balasubramanian, M. H. Metsch, P. Reddy, L. J. Rogers, N. B. Manson, M. W. Doherty, and F. Jelezko, "Discovery of st1 centers in natural diamond," Nanophotonics **8**, 1993 (2019).

[5] J. Foglszinger, A. Denisenko, T. Kornher, M. Schreck, W. Knolle, B. Yavkin, R. Kolesov, and J. Wrachtrup, "TR12 centers in diamond as a room temperature atomic scale vector magnetometer," npj Quantum Inf. **8**, 65 (2022).
